# Dosimetric comparison of different radiation techniques (IMRT vs. 3-dimensional) of the “true” (deep) ano-inguinal lymphatic drainage of anal cancer patients

**DOI:** 10.1186/s13014-018-1174-z

**Published:** 2018-11-22

**Authors:** Hendrik Dapper, Markus Oechsner, Christoph Hirche, Stefan Münch, Christina Sauter, Kai Borm, Jan C. Peeken, Stephanie E. Combs, Daniel Habermehl

**Affiliations:** 1 Department of Radiation Oncology, Klinikum rechts der Isar, TU München, Ismaninger Str. 22, 81675 Munich, Germany; 2 Department for Hand-, Plastic and Reconstructive Surgery, Burn Centre, BG-Trauma Centre Ludwigshafen/Rhine, University of Heidelberg, Ludwig-Guttmann-Str. 13, 67071 Ludwigshafen, Germany; 3 Institute for innovative Radiotherapie (iRT), Helmholtz Zentrum München, Ingolstädter Landstr. 1, Neuherberg, Germany; 4 Deutsches Konsortium für translationale Krebsforschung (DKTK), Partner SiTe Munich, Munich, Germany

**Keywords:** Anal Cancer, Ano-inguinal lymphatic drainage, Dosimetric quantification, IMRT

## Abstract

**Introduction:**

The ano-inguinal lymphatic drainage (AILD) is located in the subcutaneous adipose tissue of the proximal medial thigh. Currently, there are no recommendations for an inclusion of the ‘true’ AILD in the clinical target volume (CTV) of definitive chemoradiation for anal cancer patients. To estimate the relevance of inguinal recurrence, we compared the incidental dose to the AILD in anal cancer (AC) patients who were treated either with Volumetric Arc Therapy – Intensity Modulated Radiation Therapy (VMAT-IMRT) or conventional 3D-radiation technique.

**Methods:**

One VMAT-IMRT-plans and one 3D-plans were calculated on the same target volumes and identical dose prescription in ten patients. We defined the volume of the AILD on the planning CT-scans based on the information of new fluorescence methods. Furthermore, we defined several anatomical subvolumes of interest inside the AILD. We examined and compared absolute and relative dosimetric parameters of the AILD and different anatomical subunits.

**Results:**

The Dmean of the AILD was 40 Gy in the 3D-group and 38 Gy in the IMRT-group. Dmean and Dmedian as well as the V30Gy of the AILD and all subvolumes of the caudal AILD were significant higher using 3D-RT compared to IMRT. Even though the absolute differences were small, in the caudal aspect of the ano-inguinal lymphatic drainage the V30Gy could be more than 10% less with VMAT-IMRT.

**Conclusions:**

3D-RT was slightly superior to IMRT in terms of dose coverage of the AILD. However, the absolute differences were very small. Some relevant caudal parts of the AILD received an insufficient dose for treating potential micrometastases. Particularly in high-risk situations, this may lead to inguinal recurrence and therefore the true deep AILD should be included into the target volume in high risk patients.

## Purpose

A combined chemoradiation (CRT) protocol using IMRT is the standard treatment regimen for anal cancer [1, 2]. The anatomical definition of the lymphatic drainage of the anus to the inguinal region is complex, and the literature on this is inconsistent. In particular, the ano-inguinal lymphatic drainage (AILD) pathway has long been undetectable with traditional lymphangiography due to very small lymphatic canals [3]. Therefore, the AILD has not been properly taken into consideration for a long time, and inclusion of the elective CTV of the AILD is not recommended by standard contouring guidelines. Furthermore, CTV-recommendations are generally based not on the “anatomical” drainage but on the incidence of nodal metastases in a particular site [4, 5]. In the last years, new fluorescence-imaging methods have been developed. Those help to define the area of the AILD in real-time and transcutaneously [6, 7]. We recently published an analysis in which, for the first time, surgeons and radiation oncology specialists, presented a clearly defined anatomically area for the “true” AILD based on fluorescence-imaging method. We evaluated the dose distribution of VMAT-IMRT-treated anal cancer patients to this defined volume, showing that, in particular, the caudal areas of inguinal lymphatic drainage did not receive an adequate prophylactic dose for micrometastatic spread [8].

The clinical relevance of inguinal lymph nodes in anal carcinoma is not to be overlooked. Especially among patients with higher T-staging (T3 - T4) and those with uninvolved inguinal nodes at the time of diagnosis, who did not receive elective radiation to the groin, the inguinal recurrence rates can exceed 30 % [9–13]. If elective 3D-radiation therapy to the groin was performed without inclusion of the AILD, inguinal lymph node relapse would be considered quite rare. In an analysis of 167 patients treated between 1996 and 2004, Das et al. reported only one inguinal relapse (0.6%) in a patient with history of ipsilateral inguinal nodal involvement [14]. The risk of inguinal lymph node recurrence in IMRT treated patients is difficult to assess, as there are just a few studies demonstrating patterns of recurrence. However, even if the inguinal relapse rate in the study with most patients (106) was low (about 4%), the results indicate that using IMRT could lead to increased numbers of recurrence, especially if inguinal lymph node metastases were present at diagnosis (3 of 4 cases with relapse) [15]. Compared to 3D-techniques and field arrangements, due to steep dose gradients, IMRT offers the opportunity of dose-sparing to organs at risk (OAR) at similar loco-regional control rates [16, 17]. Even though IMRT is the recommended standard by now, using this new technique we have already experienced marginal misses in head and neck and also ano/rectal cancer patients due to misunderstandings of anatomical conditions (e. a. perirectal, presacral) [18–20].

Inguinal recurrence in IMRT-treated patients occurs mostly in patients who already had positive inguinal nodes at diagnosis. This fact raises the question, whether recurrence is due to insufficient dose to inguinal macroscopic involved nodes or an insufficient dose to potential micrometastases in AILD. To prove the impact of radiation techniques, we compared IMRT- versus 3D-techniques in anal cancer patients regarding dose distribution to the AILD. Our aim was to estimate whether the change to IMRT as the new standard technique for anal carcinoma patients could have led to insufficient dose for the treatment of micrometastases in the area of the AILD.

## Methods

### Patient selection and radiation technique details

We selected ten patients with a diagnosis of anal cancer and clinical not involved inguinal nodes who were already treated with primary chemoradiation protocol between 2012 and 2017. We prospectively generated a standardized elective clinical target volume for anal cancer as recommended by RTOG [5] on the original planning CT scan with 3 mm slice thickness for each patient. All patients were in prone position. Subsequently, we calculated two plans for each individual, one for volumetric arc therapy (VMAT) and one 3-dimensional (3D)-plan. All plans were created for a Varian Clinac® DHX linear accelerator (Varian Medical Systems, Palo Alto, CA, USA). Dose prescription for both radiation techniques was 36 Gy (1.8 Gy single dose) to PTV1, which included the primary tumor region (PTR), the elective pelvic lymph nodes and the inguinal nodes and subsequently 14.4 Gy (1.8 Gy single dose) to PTV2, which includes the PTR and the elective pelvic nodes without inguinal lymph nodes. Aim was, to cover the primary tumor site and the pelvic lymph nodes with a total dose of 50.4 Gy and the inguinal lymph nodes with a total dose of 36 Gy (single dose 1.8 Gy). Dose constraints for organs at risk (OAR) (rectum, sigmoid, small bowel, femoral head left & right, penis/scrotum or vagina/vulva, skin of AILD, urinary bladder) orientated on Quantitative Analyses of Normal Tissue Effects in the Clinic (QUANTEC) [21].

For VMAT, regularly 3 arcs in the main plan (PTV1) and 2 arcs for the boost plan (PTV2) (6 or 15 MV) were used. The dose was prescribed to the median of the PTV (ICRU83).

For 3D-radiation-plans, we utilized 6–12 fields with wedges (15–45°). Main fields were planned from posterior and lateral. Segment fields were used to improve dose coverage and dose homogeneity. The 3D planning was done according to the technique used in the past. Analogous to VMAT, the dose was prescribed to the median of the PTV (ICRU83). For both techniques we used Eclipse 13.0 Treatment Planning System (Varian Medical Systems, Palo Alto, CA, USA) for contouring and dose comparison.

### Definition of the AILD

**Fig. 1:**
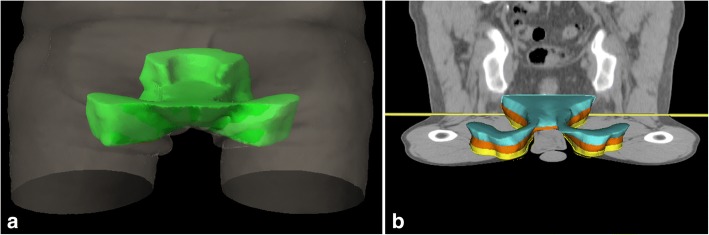
The ano-inguinal lymphatic drainage (**a**) consist of a dorsal/cranial (ischiorectal fossa) and a ventral/caudal part (subcutaneous tissue from the anus to inguinal site). The caudal part is currently not included into the elective CTV of the recommendations by RTOG. For dose comparison we divided the caudal part in three different Levels (**b**), each with a longitudinal extension of 1 cm

We defined the AILD on each patient almost identical to our previous AILD-fluorescence-study (Fig. 1a) [8]. The cranial border of the AILD (the upper ischioanal fossa) was the origin of the levator ani muscle. The caudal demarcation was defined 3 cm below the lower end of the anal canal. On inguinal site, the ventral and medial demarcation was the skin of the medial thigh; dorsal was the connection line of the dorsal edge of the gluteal muscles. The lateral demarcations were the adductor muscles (anal) or the medial edge of the sartorius or iliopsoas adductor muscles (inguinal).

In our previous study we demonstrated, that especially the caudal parts of the AILD were insufficiently covered by the 30 Gy isodose in IMRT-treated patients [8]. In contrast, the cranial parts of the AILD (ischioanal fossa) were adjacent to the anal canal and usually covered by higher doses. The interesting part of the AILD begins where the first subcutaneous connective tissue connection from the area of the anus to the inguinal lymph nodes below the pubic bone exists. This connection is not included into the CTV in various established contouring guidelines [5, 22, 23]. Therefore, we also compared the dose of different subvolumes of the AILD: We divided the AILD into a cranial part (AILDcranial = ischiorectal fossa) and a caudal part (AILDcaudal = first 3 cm below the anal verge). Furthermore, we divided the AILDcaudal into three different levels, each of 1 cm longitudinal extension (Fig. 1b).

### Dosimetric evaluation

We compared absolute (Dmean, Dmedian, D98%, D2%) and relative dose parameters (V10-V50) of the AILD for both radiation techniques. In addition, we analyzed different dose parameters to the AILDcranial, AILDcaudal and Level1, Level2 and Level3, as represented in the dose-volume histogram.

For all dose parameters of the OAR, a two-sided Wilcoxon test was performed with SPSS 25.0 (SPSS Inc., Chicago, IL, USA) to identify significant differences between the plans for several dose parameters. A *p*-value < 0.05 was considered to indicate statistical significance.

## Results

### Patient characteristics

Nine of the ten patients had anal canal cancer, whereas one patient had a T2 tumor of the anal margin. There were two patients with T1, four patients with T2 and two patients with T3 disease. Lymph node involvement could be found in four patients. All positive nodes were located in pelvic lymph nodes above the primary tumor. In both groups the PTV was well covered by the prescription dose of 36 Gy (PTV1) and 14.4 Gy (PTV2). There was no statistically significant difference between these parameters (*p* = 0.912). The measured volume of the AILD was 943 cc. Dose constraints for organs at risk (OAR) orientated on Quantitative Analyses of Normal Tissue Effects in the Clinic (QUANTEC) were respected for both radiation techniques.

### Dosimetric analysis of the whole volume of the AILD

The dose parameters of the AILD hardly differed between the two irradiation techniques (Table 1). Both, the Dmean and the Dmedian of the AILD were significant lower using IMRT compared to 3D-RT (*p* = 0.008; 9 = 0.017), even if the absolute differences were just 1.8 Gy for Dmean and 3.3 Gy for Dmedian. Ninety-eight percent of this volume was covered by 34 Gy (IMRT) and 37 Gy (3D) (*p* = 0,075). There were no relevant differences in D98% however, there was a small but significant difference in the D2% (VMAT: 51.8 Gy, 3D-RT: 53.2).

**Table 1:** Dose statistics of the ano-inguinal lymphatic drainage (AILD) with two different radiation techniques

Technique					
	Dose (Gy)				
	Dmean	Dmeadian	D98%	D2%	
3D-RT	40,2	44,8	12,5	53,2	
IMRT	38,0	41,5	11,6	51,8	
*p*-value	*0,008*	*0,017*	*0,441*	***0,007***	
	Volume (%)				
	V10Gy	V20Gy	V30GY	V40Gy	V50Gy
3D-RT	87,1	88,5	78,8	61,0	31,3
IMRT	95,6	85,3	73,1	54,2	26,9
*p*-value	*0,735*	***0,021***	***0,011***	***0,008***	*0,066*

*RT* radiation therapy, *Gy* gray, *3D* 3 dimensional, *IMRT* intensity modulated radiation therapy

Boldface indicates values that are statistically significant

Only in low dose range, the IMRT group had a non-significant higher coverage of the AILD (96%) compared to 3D-radiation (87%). With higher doses (V20Gy-V50Gy), more volume of the AILD was covered in the 3D-group, though the differences never exceeded 6 %. The V10-V40Gy were significantly higher with 3D conformal technique (*p* = 0.008–0.021). Eighty-nine percent of the AILD was covered with 20 Gy, 79 % with 30 Gy and 61 % with 40 Gy using 3D-RT, whereas 86 % of the AILD was covered with 20 Gy, 73 % with 30 Gy and 54 % with 40 Gy using IMRT.

### Dosimetric analysis of different subvolumes of the AILD

With a subdivision into different regions of interest, the AILD can be examined more precisely (Table 2). The cranial aspect of the AILD (AILDcranial), which largely corresponds to the ischio-rectal fossa, encloses the anal canal and thus the PTV. Unsurprisingly, here the Dmean and Dmedian almost reached the prescription dose of 50.4 Gy regardless of the radiotherapy technique, and 98 % of the cranial volume was covered by 34 Gy (IMRT) and 37 Gy (3D) (*p* = 0,075). Also the clinical relevant V30Gy reached almost 100 % of the volume.

**Table 2:** Dose statistics of different subvolumes inside the ano-inguinal lymphatic drainage (AILD) with two different radiation techniques

Structure	Technique	Dose (Gy)				Volume (%)
		Dmean	Dmeadian	D98%	D2%	V30Gy
AILDcranial	3D-RT	49,0	50,7	36,9	53,3	98,7
	IMRT	47,3	49,5	33,6	51,9	98,5
	*p-value*	***0,008***	***0,008***	*0,075*	***0,008***	*0,236*
AILDcaudal	3D-RT	34,7	35,4	11,3	52,3	66,5
	IMRT	32,1	32,0	10,7	51,4	57,0
	*p-value*	***0,011***	***0,011***	*0,514*	***0,018***	***0,011***
Level1	3D-RT	41,2	42,9	22,4	52,5	85,3
	IMRT	39,5	40,4	21,6	51,6	81,0
	*p-value*	***0,013***	***0,015***	*0,285*	***0,011***	*0,110*
Level2	3D-RT	36,5	37,4	15,4	51,9	73,4
	IMRT	33,8	33,7	15,6	51,4	62,6
	*p-value*	***0,011***	***0,012***	*0,722*	*0,183*	***0,008***
Level3	3D-RT	26,3	25,0	9,9	48,5	36,0
	IMRT	22,3	20,1	9,5	44,4	25,2
	*p-value*	***0,011***	*0,015*	*0,813*	*0,173*	***0,011***

*RT* radiation therapy, *AILD* ano-inguinal lymphatic drainage, *Gy* gray, *3D* 3 dimensional, *IMRT* intensity modulated radiation therapy

Level1: 1 cm of AILD caudally of the anal verge, Level2: 1-2 cm of AILD caudally of the anal verge, Level3: 3 cm of AILD caudally of the anal verge

Boldface indicates values that are statistically significant

The interesting region is the caudal part of the AILD (AILDcaudal), which cranially starts at the level of the anal verge and falls below 3 cm of the anus and inguinal nodes. Larger shares of this region are below or between the target volumes proposed by the RTOG. This is also reflected in the dose parameters. The Dmean and Dmedian were 32–35 Gy, independently of the radiation techniques, with slight but significantly higher values using 3D-radiotherapy. Ninety-eight percent of the volume was just covered by 11 Gy (both groups). Only a bit more than half of volume in the IMRT-group and two third of the volume in the 3D-group were covered by 30Gy (*p* = 0.011). The most cranial level (Level1) of AILDcaudal was partly overlapping with the PTV of the primary tumor and of the elective inguinal target volume in all patients. Here, the Dmean and Dmedian in both groups were at least 40 Gy and did not differ much from each other, although the slightly higher values in the 3D-group were statistically significant. Also Level2, which represents the volume which is 1-2 cm caudally of the anal verge, was partly covered with the PTV, which is shown in the D2% > 51 Gy in both groups. The Dmean and Dmedian, which were both significantly higher with 3D-RT, exceeded 33 Gy with IMRT and 36Gy with 3D-RT. Compared to Level1, the V30Gy was 18 % less using IMRT (63%) and 12 % less using 3D-RT (73%). The huge dose drop appeared starting 2 cm below the anal verge (Level3). The minimal dose (D2%) fell below 50 Gy. The Dmean and Dmedian were smaller 30Gy for either technique. Only one third of Level3 received 30 Gy utilizing 3D-RT, whereas the V30Gy was just 25 % with IMRT.

## Discussion

In the current study we were able to show that the Dmean and Dmedian as well as the V30Gy of the AILD and all subvolumes of the caudal AILD were significantly higher using 3D-RT compared to IMRT. Even though the absolute differences were small in the caudal aspect of the ano-inguinal lymphatic drainage, the V30Gy could be more than 10 % less with VMAT-IMRT. With both techniques a relevant volume of the AILD was not covered by 30 Gy. The results for the whole volume of the AILD were similar to our study in which we retrospectively analyzed the dose to the AILD in IMRT-treated patients [8]. Although the dose prescription in this study was slightly different, the Dmean was 41 Gy and V30Gy was 76 % compared to 38 Gy and senventy-3 % in the present study.

### Interpretation of dose distribution in context with anatomical conditions of the AILD

We chose the V30Gy as an important dose parameter because RTOG98–11 used 30.6 Gy for elective irradiation of the groin in patients with uninvolved inguinal nodes and therefore should be considered as sufficient for treatment of micrometastases. The V30Gy of the AILD was significant higher for 3D-RT (79%) than for IMRT-treated patients (73%). However, in some critical areas of the caudal AILD (Level2), the differences reached more than 10 % (73.4% versus 62.6%).

The direct drainage from the anus to inguinal site is on ventral site of the perineal pouch. Below the pubic symphysis, branches of the pudendal vessels and the first fat tissue components of the ischioanal fossa reach the external genitalia following the adductor muscles (lateral) and the skin (medial) [24]. We chose this area as the first level of our anatomically detailed breakdown of dose distribution (Level1). The anus and inguinal site at this level were covered by the previous PTV and received adequate dose with both radiation techniques. For this critical level, the V30Gy was 85 % with 3D-RT and only 4 % lower (81%) with IMRT. The absolute differences are indeed small and it would be daring to assume that these small differences increase the risk of inguinal recurrences.

**Fig. 2:**
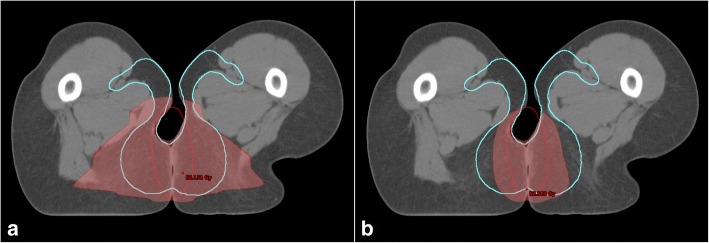
Axial CT slide 1.5 cm below the lower end of the anal canal in one patient. 30 Gy is represented in colorwash (red area). In this case, about 75% of ano-inguinal lymphatic drainage (cyan) is covered with 3D-RT (**a**) and just about 50% is covered using IMRT (**b**)

Around 1 cm below this level we defined Level2. The differences in dose distribution between the two techniques increased. Figure 2 shows the 30 Gy isodose (color wash) in a selected patient using 3D-RT (2a) or IMRT (2b). The caudal parts of the AILD were properly covered with 3D-RT, whereas in IMRT the V30Gy was more conformal surrounding the PTV, and less volume of the AILD was covered. Ultimately, this region could be the cause of regional recurrences in some patients with high risk tumors treated with IMRT. With immune-fluorescence methods we were previously able to show the AILD to fall about 3 cm below the level of the anal verge. This level (Level3) is the most caudal potential lymphatic drainage of the anal verge and unlikely to contain micrometastases in low risk tumors of the anal canal. Since inguinal recurrences rarely occur 3 cm below the primary tumor, we do not expect this level to be responsible for inguinal relapse in the vast majority of cases. Nevertheless, in high risk anal cancer, especially for big tumors (≥ T3) of the anal verge with a high number of positive inguinal and pelvic lymph nodes at diagnose, this part of the AILD could be of clinical relevance. The change from 3D-RT to IMRT might have an impact regarding inguinal recurrence in those patients.

### Clinical relevance of elective radiation of lymphatic-drainage

The most common grade 3 or 4 toxicity in the two big prospective trials (2D- or 3D-techniques) was radio dermatitis. In both trials (RTOG98–11 and ART II) grade ≥ 3 skin toxicity was 48 % in the mitomycin-based arm [1, 2]. Scher et al. summarized 8 IMRT-studies which demonstrated skin toxicity after radiation of anal cancer patients (*n* = 39–78) [25]. Dose to the inguinal lymph nodes was mainly 45 Gy. Grade ≥ 3 skin toxicity ranged from zero to 42 % (mean: 23%). With IMRT-techniques ≥3, skin toxicity was significantly reduced in RTOG-0529 (23%), whereas in other IMRT-studies it could reach 69 % [15, 26, 27]. Because of the fact that the critical part of the ano-inguinal lymphatic drainage is located subcutaneously on the medial thigh, we would expect an increase in this relevant toxicity. Furthermore, the genito-urinary side effects might increase [28].

In order to ascertain the clinical benefit of inclusion of the AILD into the CTV, the number and pattern of recurrence are important. Overall, the data on IMRT-treated patients and explicit inguinal recurrences and patterns of spread are less well explored. In the RTOG-0529-study, loco regional failure was 13 % after 4 years. The Study did not show any detailed differentiation of the site of failure, though [17]. Only Tomasoa et al. provided detailed data regarding the number and patterns of inguinal recurrence. In a retrospective analysis of 106 patients treated by SIB-IMRT, four patients had inguinal relapse (4%) [15]. Three out of these four patients had initially involved inguinal nodes. A total dose to lymph nodes of 49.5 à 1.5 Gy was given, which means a higher biological dose than in conventional techniques. The authors (Tomasoa et al.) compared this to similar results of Wright et al. (3-field technique) and mentioned that more conformal techniques (IMRT) with higher total dose might not reduce inguinal relapse [15, 29]. This indicates that microscopic disease in the AILD but not the lymph nodes themselves might be responsible for inguinal relapse. Another important finding of these studies was that none of inguinal recurrences occurred below approximately 1 cm of the level of the anal canal. Wright et al. however, could show that inguinal recurrences arise up to 3 cm below the anal verge (conventional technique) [29]. Up to this date, insufficient data concerning inguinal recurrence in anal carcinoma is available. However, there are a few cases of inguinal relapse even in inguinal-treated patients. To balance the relevant skin toxicity with the risk of inguinal relapse in high risk tumors (T3-T4, inguinal involvement at diagnosis, anal verge), an inclusion of the AILD into the target volume could be useful.

## Conclusion

3D-RT was slightly superior to IMRT in terms of dose coverage of the AILD. However, the absolute differences were very small. Some relevant caudal parts of the AILD received an insufficient dose for treating potential micrometastases. Particularly in high-risk situations, this may lead to inguinal recurrence and therefore the true deep AILD should be included into the target volume in high risk patients.

## Abbreviations

2D/3D: 2/3 dimensional
AILD: Ano-inguinale lymphatic drainage
CRT: Chemoradiation therapy
CTV: Clinical target volume
E. a.: For example
Gy: Gray
IMRT: Intensity modulated radiotherapy
LD: Lymphatic drainage
OAR: Organs at risk
PET: Positron emission tomography
PTR: Primary tumor region
PTV: Planning target volume
RT: Radiation therapy
SIB: Simultaneously integrated boost
VMAT: Volumetric modulated arc therapy
